# Design of experiments to investigate multi-additive cellulose nanocrystal films

**DOI:** 10.3389/fmolb.2022.988600

**Published:** 2022-11-04

**Authors:** Patrik Nilsson, Åsa Engström, Joice Jaqueline Kaschuk, Jaana Vapaavuori, Arvid Larsson, Tiffany Abitbol

**Affiliations:** ^1^ Bioeconomy, RISE Research Institutes Sweden, Stockholm, Sweden; ^2^ Department of Bioproducts and Biosystems, School of Chemical Engineering, Aalto University, Espoo, Finland; ^3^ Department of Chemistry and Materials Science, Aalto University, Espoo, Finland; ^4^ Centre for Mathematical Sciences, Faculty of Engineering, Lund University, Lund, Sweden; ^5^ Institute of Materials, EPFL, Lausanne, Switzerland

**Keywords:** cellulose nanocrystals, self-assembly, films, design of experiments, additives, machine learning

## Abstract

Cellulose nanocrystal (CNC) suspensions can self-assemble into chiral nematic films upon the slow evaporation of water. These films are brittle, as indicated by their fracturing instead of plastically deforming once they are fully elastically deformed. This aspect can be mediated to some extent by plasticizing additives, such as glucose and glycerol, however, few reports consider more than one additive at a time or address the influence of additive content on the homogeneity of the self-assembled structure. In this work, design of experiments (DoE) was used to empirically model complex film compositions, attempting to relate additive concentrations in dilute suspension to film properties, and to understand whether outcome specific predictions are possible using this approach. We demonstrate that DoE can be used to predict film properties in multi-additive systems, without consideration given to the different phenomena that occur along the drying process or to the nature of the additives. Additionally, a homogeneity metric is introduced in relation to chiral nematic organization in CNC films, with most of the additive-containing compositions in this work found to reduce the homogeneity of the self-assembly relative to pure CNC films.

## 1 Introduction

The property space for nanocellulose-based films is large, especially when additives are included to tailor properties like optics or elasticity (Habibi et al., 2010; Kaschuk et al., 2022). Inevitably, a large property space requires a large experimental space to map. Design of experiments (DoE) can be used to optimize the information gained per experiment, effectively minimizing experimental load while maximizing meaningful results. Combining DoE with machine learning tools can be a powerful way to delineate the large property space accessible in nanocellulose films and to enable general conclusions on the influence of different additives on film properties. Here, we use this approach to study cellulose nanocrystal (CNC) films containing additives.

CNCs are nanoparticles typically isolated by the acid hydrolysis of native cellulose from different origins, such as wood, cotton, tunicate, and bacteria (Revol et al., 1998; Habibi et al., 2010). CNC suspensions organize into chiral nematic liquid crystalline phases, defined by a half-pitch, which gives the distance along the helical axis traversed in a 180° rotation of the average CNC orientation (Dong et al., 1996). Chiral nematic organization can be captured in films by evaporation (Revol et al., 1998), however the continual removal of water during film formation is non-equilibrium and not all films exhibit clear chiral nematic assembly. In the end, the overall mesoscale uniformity of a given film depends on different factors, most critically colloidal stability, with highly organized films as well as intermediate or mixed phase films reported in the literature (Kaschuk et al., 2022). Films are structure colored if the chiral nematic pitch coincides with visible light wavelengths, but can also be transparent or hazy (Kaschuk et al., 2022). Different additives have been included in CNC films to modify optical and tensile properties, often by influencing self-assembly and film formation (Mu and Gray, 2014; Bardet et al., 2015; Guidetti et al., 2016; de La Cruz et al., 2018; Xu et al., 2018; Adstedt et al., 2020; Jin et al., 2021).

In this work, the influence of plasticizing additives on CNC films is explored using a DoE framework to map the experimental space and to assess the predictive power of multivariate models in relating suspension properties to film properties. CNC-based films containing up to four different plasticizers (glucose, glycerol, locust bean gum, and glucuronic acid) were prepared, with the sample size set designed to allow the construction of non-linear models. Other studies have used DoE similarly but in the context of CNC preparation, usually to optimize hydrolysis conditions to favor a higher CNC yield or specific properties, such as length and surface charge density (Wang et al., 2012, 2014; Vanderfleet et al., 2018).

Generally, the application of machine learning algorithms requires enough training samples to build a model and an appropriate assessment of model fit and possible overfit, which increases in risk with larger models (Sejnowski, 2018; Bhasin, 2020). For larger models, such as neural networks and random forests, the risk of overfitting is usually addressed by testing the model on data not used in its training, requiring that the model itself be built of sufficient samples for patterns to be discernible above the levels of experimental noise (Sejnowski, 2018; Bhasin, 2020). Related work aimed at optimizing the properties of additive-containing cellulose nanofibril films used neural networks and random forests that were trained on few samples and tested for model overfitting by excluding training samples instead of introducing new samples (Özkan et al., 2018, 2019). Additionally, the use of mean absolute percentage errors (MAPE) as an error metric to evaluate model fit may be problematic for homoscedastic samples (Özkan et al., 2018, 2019).

In our work, tensile and optical properties were studied using multiple linear regressions built from 80 distinct samples, however a smaller sample size was used for regressions related to chiral nematic organization since fewer samples exhibited this feature. From the starting point of either linear, interaction, or quadratic models, model coefficients were evaluated and removed if not significantly different from zero. This process was repeated iteratively for the three model types, and when no additional coefficients could be removed, model accuracy was gauged using adjusted *R* ^2^ values, and the model with the highest adjusted *R* ^2^ was chosen, usually a linear model. In addition to a small model size from the start, the iterative approach used in the multiple regressions further reduced the size of the model and the risk of overfitting. Logistic regressions and random forests were also used, but here fit was evaluated with a 5-fold cross validation and overfit with test samples (test set was 15% of training set).

The development of models that can predict the properties of nanocellulose-based films is likely to prove important for expanding the application range of these materials. Significantly, the implementation of data-based models does not require a mechanistic understanding, which can be challenging in complex, multi-component systems, but nonetheless their results can hint at important interactions. In this study, the models were able to predict with reasonable accuracy tensile strength index, Young’s modulus, and whether or not films were chiral nematic, but were less predictive when the effect of the additives was low in relation to experimental error, such as for transparency, haze, and strain at break. A homogeneity metric was applied to chiral nematic films to capture the loss in reflection intensity and increase in peak breadth that were often encountered in additive-containing films, addressing that additive inclusion is not always without cost. Finally, we suggest that the general approach used in this work can be extended to other mixed systems, to identify areas in of interest in a given property space, optimized compositions, and patterns that inform mechanistic understanding, all of which were done in this study using different models.

## 2 Materials and methods

### 2.1 Materials

Spray dried CNCs from CelluForce, and D-glucose, D-glucuronic acid, glycerol, locust bean gum (LBG; approximately 310 kDa), polyvinyl alcohol (PVA; 89–98 kDa, 99 +% hydrolyzed), dextran, guar gum, and gum karaya from Sigma Aldrich were used as received. Milli-Q water was used in all instances.

### 2.2 Methods

#### 2.2.1 DoE

A D-optimal design was constructed in MODDE and used to determine the experimental levels to test. The testing space was defined broadly to be between 0 and 20 wt% of any given additive, with the additional constraint of the total additive concentration not exceeding 30 wt%.This analysis resulted in the design matrix presented in Supplementary Table S1, which also considered interaction effects. In addition to this design, six smaller designs were constructed to address specific questions about the additives. The design in Supplementary Table S2 was used to elucidate whether glucuronic acid induces CNC aggregation at higher concentrations, in Supplementary Table S3 to identify the highest concentration of locust bean gum that can be tolerated for chiral nematic organization and whether this concentration is shifted by the presence of glucuronic acid. Supplementary Tables S4, S5 are designs that also included NaCl to better understand the effects of glucuronic acid on chiral nematic self-assembly and whether its effects could be muted or made more glucose-like by the addition of salt to screen charge, and the design in Supplementary Table S6 was used to provide a final test of the models. The design in Supplementary Table S7 was used in a pre-study to investigate potential additives of interest.

### 2.3 Preparation of cellulose nanocrystal suspension, stock additive solutions, and mixtures

A stock CNC suspension at 2 wt% was prepared by combining commercial spray dried CNC powder with water and mixing overnight with a mechanical overhead mixer. Next, the mixture was dispersed by 1 pass through a microfluidizer (M110-EH) at 1700 bar (200 μm and 100 µm chambers), followed by vacuum filtration (Munktell 3) to remove any dust or larger particulates. The concentration of the CNC suspension was then verified gravimetrically by drying in an oven at 105°C. Stock solutions at 1 wt% were prepared from glucose, glucuronic acid, and glycerol by dilution in water and magnetic stirring. The LBG stock solution was prepared at 0.5 wt% by adding the LBG powder to water and heating the mixture to 70°C for 24 h with magnetic stirring. The solution was then vacuum filtered (Munktell 3) twice to remove agglomerates. After filtration, the solid content was determined gravimetrically (approx. 0.2 wt%). According to the experimental designs, the stock solutions and CNC suspension were combined with water to a final CNC concentration of 0.35 wt%, with locust bean gum, glucuronic acid, glucose, and glycerol included at a maximum of 20, 40, 32, and 20 wt% of the CNC content, respectively. Suspensions were similarly prepared in a pre-study that focused on the following additives: dextran, PVA, gum karaya (with and without vacuum filtration), glycerol, and guar gum.

### 2.4 Film casting

Suspensions were poured into 9 cm-diameter Petri dishes to achieve a film grammage of 30 g/m^2^. The suspensions were then slowly evaporated until dry (1–2 weeks) in a conditioned environment (23°C and 50% RH). Three films were cast from each concentration combination, with the remaining volume used for suspension characterization. Films from abovementioned pre-study suspensions were cast identically but at a target grammage of 60 g/m^2^.

### 2.5 Dynamic light scattering and zeta potential

A Malvern Zetasizer Nano ZS was used for DLS and zeta potential measurements. The additive content was 25 wt% for single additive compositions, and 12.5 wt% each for compositions containing two additives. Samples were diluted in 10 mM NaCl to 0.01 wt% for zeta-potential and 0.001 wt% for DLS and filtered through 0.45 µm PVDF syringe filters prior to measurement in triplicate. Z-average size and zeta-potential analyses assume spherical particle shape and are therefore only considered comparatively in this work.

### 2.6 Atomic force microscopy

AFM imaging was done using a Multimode eight AFM (Bruker, Nanoscope V controller) in tapping mode. The signal was processed in the Nanoscope Analysis 1.8 software by Bruker. ScanAsyst-Air cantilevers were used, with a nominal spring constant of 0.4 N/m. Dilute samples (0.01 wt%) were deposited by spin-coating onto mica substrates that were previously cleaved, spin-coated with polyallylamine hydrochloride (0.1 wt%) and rinsed with water.

### 2.7 Polarized optical microscopy

A Zeiss Axioplan microscope with crossed polarizers and a red waveplate was used visualize the self-assembly in films. POM images were used to group the different films into classes based on their distinct birefringence. The categories are listed in Supplementary Table S8.

### 2.8 Ultraviolet-visible-near-IR spectroscopy

Film reflectance (R), transmittance ( $T_{T}$ ), haze, and diffuse transmittance ( $T_{D}$ ) were measured using a Diffuse Reflectance Accessory coupled to an Agilent Cary Series UV-Vis NIR Spectrometer at 1 nm intervals between 200 and 2,500 nm. The value of these properties at 550 nm was chosen as representative of film optical properties. Transmittance at 550 nm of the different films is reported in Supplementary Table S8.

### 2.9 Tensile testing

Rectangular strips (45 mm long and 6 mm wide; seven strips per film) were die-cut using a custom stamp. The average grammage of each film was calculated from the weight of 4 strips. Using an MTS 3125 tensile tester, tensile tests were performed on the center 30 mm of all 7 strips and the strain at break ( $\varepsilon_{b}$ ), tensile strength index ( σ ), tensile stiffness index (TSI) were determined from the recorded stress-strain curves. Young’s modulus, σ , and $\varepsilon_{b}$ are presented in Supplementary Table S8. The average thickness of the films was also measured using an STFI thickness tester at five different spots (Supplementary Table S9). Films were equilibrated for several days under standard conditions (50% RH, 23°C) prior to measurement.

### 2.10 Scanning electron microscopy

Fracture cross sections of films were imaged under high vacuum with a 2 kV acceleration voltage using a Quanta 250 FEG ESEM by FEI Instruments that was coupled to a X-Max 50 mm^2^ EDS by Oxford Instruments. The detector used was an Everhart-Thornley Detector. Cross sections were produced in the tensile testing.

### 2.11 Chiral nematic homogeneity

For films exhibiting features of chiral nematic organization, the homogeneity of the assembled structure was defined as the intensity of the reflection peak (height of the peak in the reflectivity spectrum) divided by the full width at half height of the peak. Homogeneity values were then normalized to CNC content and are reported as a percentage relative to the homogeneity of a pure CNC control film.

### 2.12 Modelling

Multiple linear regressions of varying sizes were used to interpret quantitative results (mechanical and optical properties) and a logistic regression was used to interpret qualitative results (film self-assembly based on polarized optical microscopy).

(i) Quantitative models

A multiple linear regression is defined by fitting vector **B** and intercept **I** to solve Eq. 1 using the least squares method (Brereton, 2003).

$$\begin{array}{c} Y=XB+I+\varepsilon \end{array}$$ (1)

$$Y=\left[\begin{array}{c} y_{1}\\ \vdots \\ y_{n} \end{array}\right],X=\left[\begin{array}{ccc} x_{1,1} & \cdots & x_{1,m}\\ \vdots & \ddots & \vdots \\ x_{n,1} & \cdots & x_{n,m} \end{array}\right],B=\left[\begin{array}{c} k_{1}\\ \vdots \\ k_{m} \end{array}\right],\;\varepsilon =\left[\begin{array}{c} \varepsilon_{1}\\ \vdots \\ \varepsilon_{n} \end{array}\right]$$ (2)

In Eqs 1, 2, the **X-**matrix corresponds to additive concentrations and higher order terms, such as interaction and quadratic terms, the **Y**-vector represents the measured values of a given property and the ε -vector is the model error (Brereton, 2003). Eq. 2 was solved using the Python module statsmodels v0.12.2 (Seabold and Perktold, 2010).

By linearly regressing additive concentrations to the measured properties of the films ( E , $\varepsilon_{b}$ , σ , TSI, R , and $T_{T}$ , chiral nematic homogeneity, and pitch), models predicting these properties could be constructed. To achieve high predictive power and avoid overfitting, multiple linear regressions with only linear, linear and interaction, and linear, interaction, and quadratic terms were constructed. Then, in a top-down fashion, coefficients that were not significantly distinguished from zero at 95% confidence were removed and the model was reconstructed. This process was repeated iteratively until all coefficients were significantly separated from zero. The remaining model with the highest adjusted $R^{2}$ -value after this process was chosen.

(ii) Qualitative models

After careful consideration of the POM images, three categories based on the appearance of film birefringence patterns were identified. Then, each composition was assigned to a given category and logistic regressions were constructed to predict film categories from the concentrations of additives used to make the films. A logistic regression in the case of a binary response can be described by Eq. 3 (Walker and Duncan, 1967):

$$\begin{array}{c} \log\frac{p}{1-p}=\beta X \end{array}$$ (3)

where the β -vector is fitted using a linear regression, p is the probability of the response being one of two possible outcomes, and X is a vector containing the input variables (Walker and Duncan, 1967). Using scikit-learn in Python 3 (Pedregosa et al., 2011), we extended this approach to a tertiary response based on the birefringence classes. A detailed explanation of the mathematics of tertiary logistic regressions is beyond the scope of the current work but can be found elsewhere (Engel, 1988). A random forest model, which is larger, i.e., has a higher risk of overfitting but can model more fine-grained concepts than the logistic regression, was also constructed to identify whether there are phenomena in the film formation that are too complex for the logistic regression to model accurately. This was also done using scikit-learn in Python 3 (Pedregosa et al., 2011).

## 3 Results

### 3.1 Select experimental data

To better understand the interactions of the additives with the CNCs, we additionally explored select binary and trinary compositions outside of the model space. DLS and zeta-potential results of these simplified compositions are presented in Table 1.

**TABLE 1:** DLS and zeta potential results, with associated standard errors calculated at 95% confidence. For additive-containing suspensions, the total additive content was set to 25 wt% of the CNC content. For suspensions with two additives, each additive was set to 12.5 wt%.

Suspension	Z-average size (nm)	Zeta potential (mV)
CNC, pure	64.6 ± 0.3	−50.0 ± 1.2
+LBG	79.0 ± 0.7	−28.8 ± 1.8
+Glucuronic acid	65 ± 0.5	−44.6 ± 0.4
+Glucose	70.8 ± 0.6	−49.9 ± 1.3
+Glycerol	72 ± 0.5	−49.9 ± 1.3
+LBG + Glucuronic acid	93.2 ± 1.6	−31.5 ± 1.5
+LBG + Glucose	87.5 ± 2	−37.5 ± 1.0
+ LBG + Glycerol	79.6 ± 0.2	−35 ± 2

From Table 1, the z-average size and zeta potential values of a pure CNC suspension and of suspensions containing either 25 wt% glucuronic acid, glucose, or glycerol, were quite similar, ranging from 65 to 72 nm and −50 to −45 mV, respectively. The LBG-containing suspension had a somewhat larger z-average size at 79 nm and a less negative zeta potential at −29 mV. The increase in size is likely related to LBG adsorption, and the lower absolute value of the zeta potential has previously been attributed to a shift in the slipping plane due to adsorbed polymer (Hu et al., 2014). LBG in combination with glycerol had a similar z-average size as when LBG was the sole additive (even though the LBG content was halved), whereas either glucose or glucuronic acid in combination with LBG increased particle size perhaps suggesting that these additives promote LBG adsorption in a more extended conformation. In terms of the zeta potential of the bi-additive suspensions, it is difficult to parse out trends as both adsorption and solvent properties are changed. Overall, however, LBG seems to dominate the results in bi-additive suspensions, increasing particle size and decreasing the zeta-potential.

**FIGURE 1:**
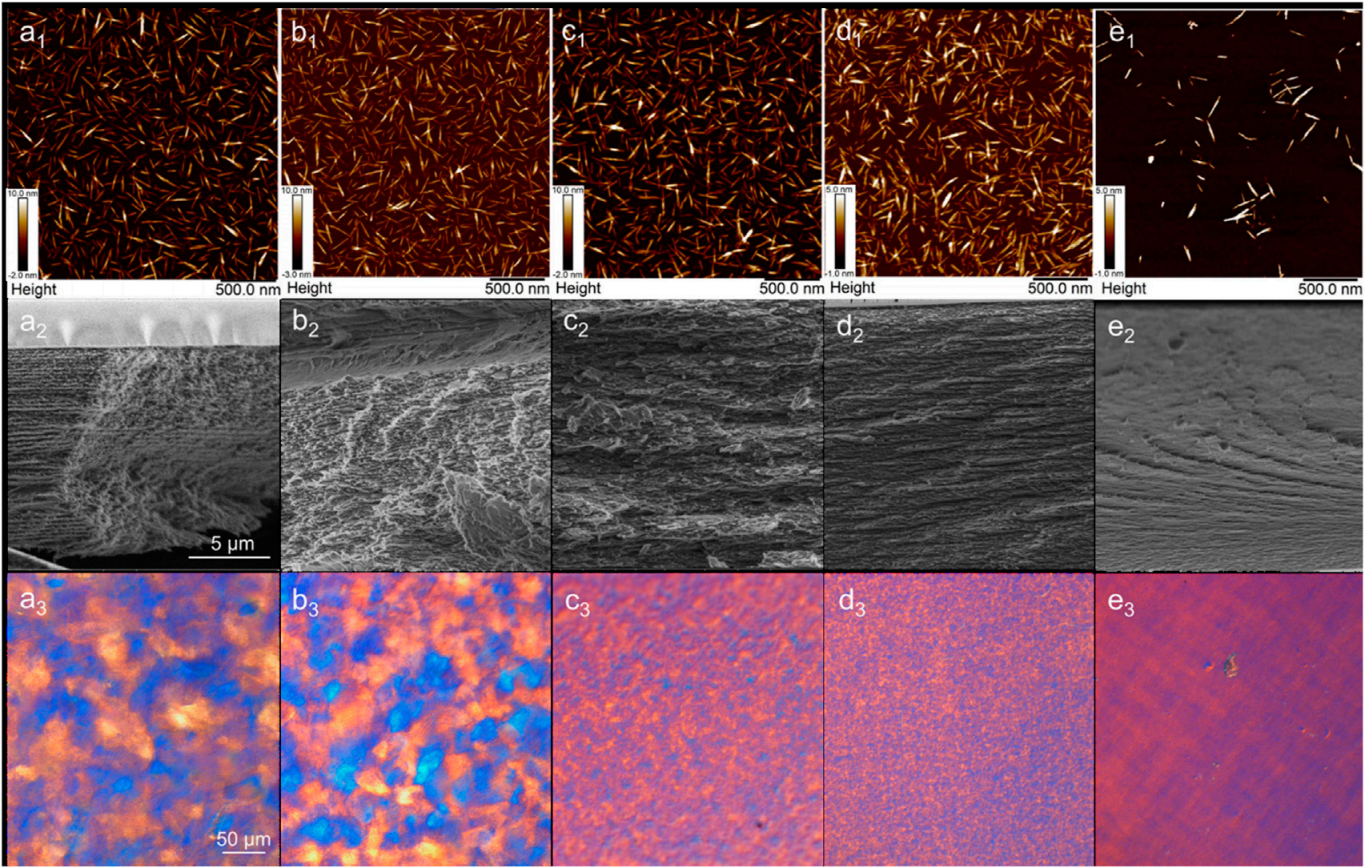
Suspension AFM (top row), cross-sectional film SEM (middle row), and film POM (bottom row) of select compositions: Pure CNC **(a_1_–a_3_)**, 80 wt% CNC and 20 wt% glucose **(b_1_–b_3_)**, 99 wt% CNC and 1 wt% LBG **(c_1_–c_3_)**, 80 wt% CNC and 20 wt% glucuronic acid **(d_1_–d_3_)**, and 80 wt% CNC and 20 wt% LBG **(e_1_–e_3_)**.

**FIGURE 2:**
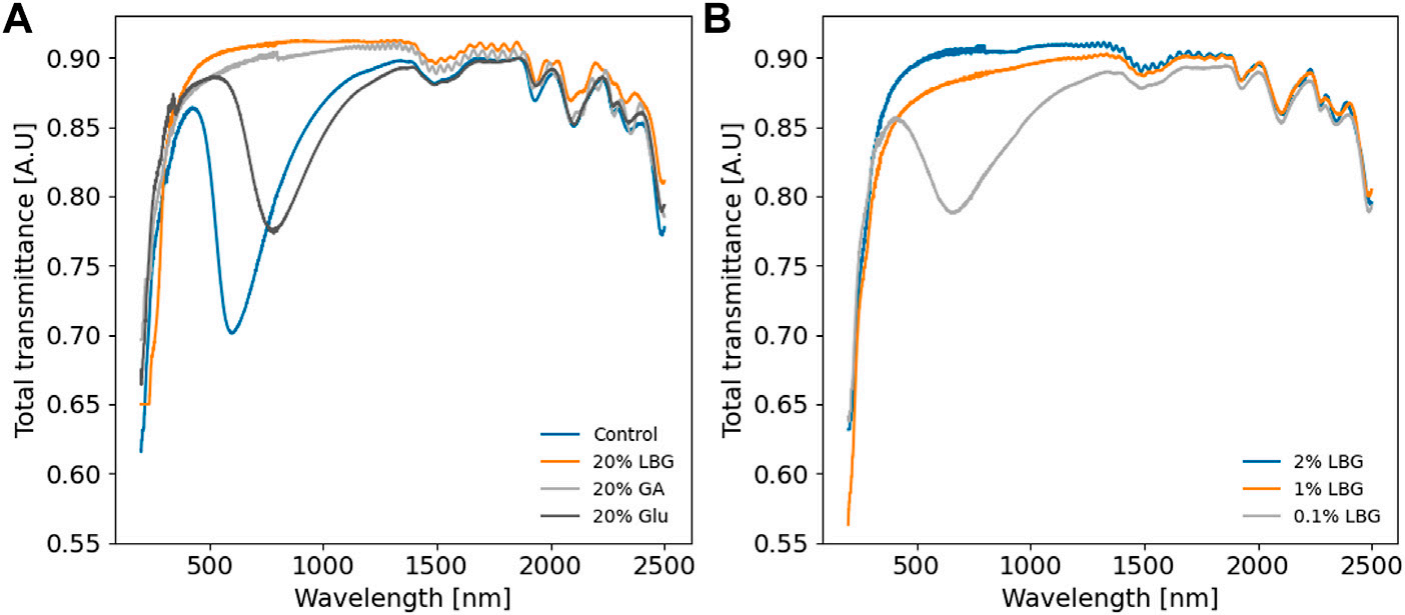
UV-vis-NIR spectra of select film compositions, at 20 wt% additive loading in **(A)** and exploring LBG limits in **(B)**. All films are similarly transparent, except for the chiral nematic films, which show a dip in total transmittance due to reflection. Glucose and glucuronic acid are abbreviated as Glu and GA, respectively. Spectra are not normalized.

**FIGURE 3:**
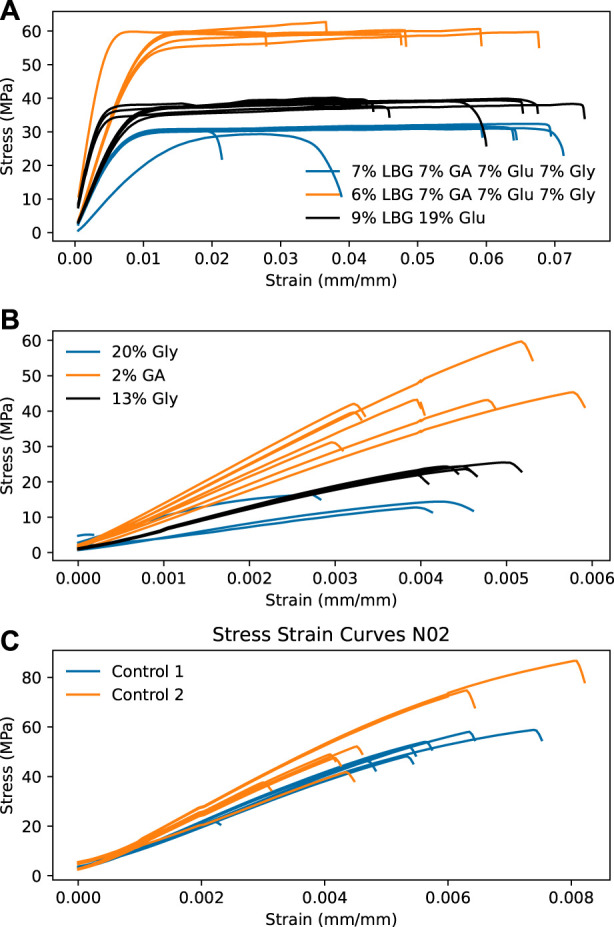
Stress-strain curves from films with the highest average $\varepsilon_{b}$ **(A)**, the lowest average $\varepsilon_{b}$ , **(B)**, and CNC controls **(C)**. Glucose, glucuronic acid, and glycerol are abbreviated as Glu, GA, and Gly respectively.

Next, select results are presented from within the model space to highlight the main features that were experimentally observed. Figure 1 shows suspension AFM, cross-sectional film SEM, and film POM. Figure 2 shows film UV-Vis-NIR spectra. Figure 3 presents tensile properties, here emphasizing compositions that produced the most significant differences from pure CNC control films.

The suspensions appear alike by dilute suspension AFM (Figure 1, top row), except for the composition containing 20 wt% LBG (Figure 1e_1_), which has substantially lower surface coverage, perhaps due to agglomeration. SEM (Figure 1, middle row) and POM (Figure 1, bottom row) show more variation across the different compositions. The 100% CNC film (Figure 1A) and the film with 20 wt% glucose (Figure 1B) show features typical of chiral nematic assembly both by SEM and POM, which is confirmed by the valley between 500 and 1,000 nm in the transmittance spectra of these films (Figure 2). Others have also observed that the ability of CNCs to form chiral nematic structures is relatively unimpaired in the presence of glucose or glycerol (Mu and Gray, 2014; Xu et al., 2018). Films with 1 wt% of LBG (Figure 1C) and 20 wt% glucuronic acid (Figure 1D) both show similarly interrupted and less organized structures, indicated by the smaller size of the birefringent domains in the POM and by an increase in cross-sectional voids in the SEM. Similar POM and SEM images found elsewhere in the literature have been interpreted as a stunted chiral nematic domain growth due to conditions that screen chiral interactions between CNCs (Abitbol et al., 2020; Jin et al., 2021). The characteristic features of a chiral nematic structure, specifically birefringent microdomains and fingerprint patterns in POM and regular arced layers in SEM (Guidetti et al., 2016; Xu et al., 2018; Bumbudsanpharoke et al., 2019), are fully absent in the film containing 20 wt% LBG (Figure 1E). Others have interpreted similar images of CNC films as indicative of a loss in chiral nematic organization tending toward nematic assembly (Walters et al., 2020).

The POM images presented in Figure 1 exemplify the different categories according to which all films produced in this work were broadly classified. We interpret the films presented in Figure 1 as existing along a continuum of organization (most clearly visualized by the POM sequence in Figure 1), visualized as a twisted chiral nematic structure that unwinds from left to right. Along this spectrum, the 100% CNC film (Figure 1a_3_) is considered “the most” chiral nematic. Next, the film compositions that yield small birefringent domains are attributed to an underdeveloped or pre-chiral nematic structure (Figure 1c_3_ and d_3_). Finally, we have compositions that result in largely monochromatic POM, sometimes with cross-hatched features, which we relate to an apparent untwisted planar assembly and the apparent absence of chiral nematicity (Figure 1e_3_). In shorthand, we refer to these structures as *CN* for chiral nematic, *SD* for small domain, and *UTP* for untwisted planar (non-specific). (The *UTP* category is not rigorously defined and is intended as a sort of catch-all for the POM images that were not classified as *SD* or *CN*.) Furthermore, it is apparent from Figures 1, 2 that not all chiral nematic films are equal in their homogeneity, an aspect which can be difficult to ascertain from POM or SEM imaging alone.

Figure 3 shows stress-strain curves of film compositions with the highest and lowest strains at break, as well as the tensile properties from two pure CNC control films. From Figure 3, the combination of multiple additives can give plastic behavior, for instance 9 wt% LBG with 19 wt% glucose gives a ten-fold increase in the strain at break compared to the control films. The highest strain at break is achieved for combinations of all four additives or of LBG and glucose, whereas the lowest strain at break occurred in films that contained one additive, either glucuronic acid, glucose, or glycerol. There are conflicting results in the literature, with some studies reporting an overall increase in strain at break with added glycerol (Xu et al., 2018), while others report increases at low glycerol loadings and decreases with higher amounts of glycerol (Rivkin et al., 2015; He et al., 2018). Interestingly, films with 6 wt% LBG and those with7 wt% of the other three additives, not only break at a magnitude higher elongation but are also as strong or stronger than pure CNC films, which may be significant for applications that require toughness. Notably however, as is seen in Figure 3, the standard deviation of the strain at break is rather large, indicating rather low repeatability due to the general brittleness of CNC-based films, even with added plasticizer.

### 3.2 Chiral nematic homogeneity

**FIGURE 4:**
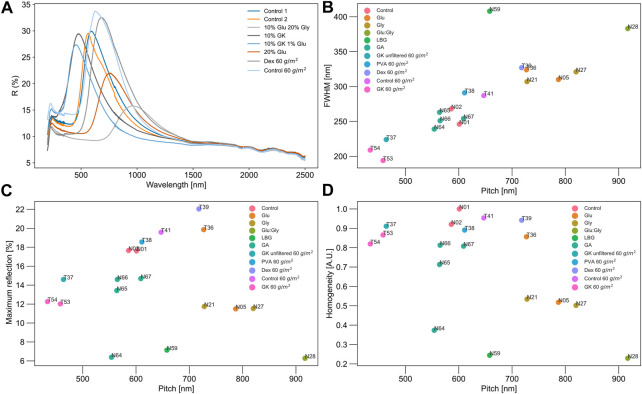
Select reflectance spectra of chiral nematic films **(A)**, full width at half-maximum of reflectance peak **(B)**, % reflected light at peak maximum **(C)**, and homogeneity **(D)**, plotted against pitch, with pitch defined as the wavelength of maximum reflection. Films containing additives outside of the design were included in this analysis to extend the data set of chiral nematic films. These films containing gum karaya (GK), dextran (Dex), or polyvinyl alcohol (PVA) had a higher grammage of about 60 $g/m^{2}$ , whereas all other films had a target grammage of 30 $g/m^{2}$ . Homogeneity was defined as the height of the reflectance peak divided by its full width at half-height, normalized to the homogeneity of the pure CNC film (N01). In **(A)**, the legend gives additive content in percentage, whereas in **(B–D)**, alphanumeric labels refer to the compositions presented in Supplementary Tables S1–S7 and color codes for the different additive/additive combinations.

Figure 4 provides a breakdown of the different chiral nematic optical parameters used to address the overall homogeneity of the self-assembly. Representative reflectance spectra are shown in Figure 4A, with the % reflected light, full width at half-maximum, and ratio of the % reflected light to the full width at half-maximum (“homogeneity”) plotted as a function of pitch in Figures 4B–D. The homogeneity metric was formulated to compare the influence of different additive combinations on chiral nematic self-assembly, coupling the effects of peak width as a proxy for domain uniformity and % reflected light as a proxy for overall chiral nematicity. Many studies that consider chiral nematic films do not address changes in full width or % reflected light, focusing instead on pitch. However, for color-based applications, the overall uniformity and intensity of the optical response across a given optical pathlength may be just as important as its color.

Three control CNC films are shown in Figure 4, N01, N02, and T41, with T41 at approximately twice the grammage as N01 and N02, exhibiting a slightly higher % reflected light at peak maximum, broader width, and similar pitch. The homogeneity of these films is similar as might be expected, reflecting approximately 20% of the incoming light. Minor variations between these films can be attributed to differences in film thickness.

Beck et al. (2013) attributed redshifts in CNC films to a general phenomenon related to greater disorder in the chiral nematic domains of longer pitch films, inferred from increases in full width at peak maximum with increasing pitch (Beck et al., 2011). The results presented in Figure 4C support this conclusion, with redder reflections coinciding with larger full widths. Similar results can be found in other publications, for instance, glycerol-containing CNC films have broader peaks, lower intensities, and redder reflections with increasing glycerol, indicative of more disordered assembly (He et al., 2018). For the redshifted films (T36, T39, N05, N21, N27, and N28), the homogeneity of N05, N21, N27, and N28 was decreased compared to the CNC control films due to peak broadening, whereas homogeneity was relatively maintained for T36 (10 wt% dextran) and T39 (10 wt% glucose). This is because T36 and T39 exhibited similar or slightly higher % reflectance compared to the control CNC films. Almost doubling the glucose content from 13 (N24) to 20 wt% (N05), decreased the % reflected light, without altering peak width very much.

Tran et al. found that film homogeneity could be significantly improved by slowing the evaporation process, with films cast from suspensions that were covered for set times during film formation exhibiting narrower peak widths and bluer peak positions the longer the coverage time (Tran et al., 2018). However, the reflectance spectra were normalized, making it difficult to address overall chiral nematic homogeneity or compare to our study directly (Tran et al., 2018). For the films in this work exhibiting a significant blue-shift (T37, T53, and T54), peak widths were narrowed, giving a relatively high homogeneity. T37, T53, and T54 contained 10 wt% gum karaya with 0, 5, and 1 wt% of added glycerol, respectively.

Finally, N59, N64-67, and T38, have a pitch close to that of the control films, but exhibit a range of homogeneities, from ∼0.2–0.9 (Figure 4D), due to either a lower percentage of reflected light or peak broadening, or both (e.g., N59 with LBG). Most additive-containing films studied in this work show a decrease in homogeneity, which becomes increasingly compromised the higher the additive content (Figure 4D), suggesting a less tidy chiral nematic assembly. However, some additives do not influence homogeneity significantly, specifically 10 wt% PVA (T38), 10 wt% dextran (T39), and 10 wt% gum karaya (T37, T53). This suggests that additions of PVA, dextran, or gum karaya at 10 wt% can be tolerated without altering CNC self-assembly. The loss in homogeneity observed with some additive/additive combinations points to a cost in terms of optical performance when plasticizers are included in films. (Other costs may relate to additive migration and/or aging behavior, but these are out of the current scope.)

### 3.3 Quantitative models

**FIGURE 5:**
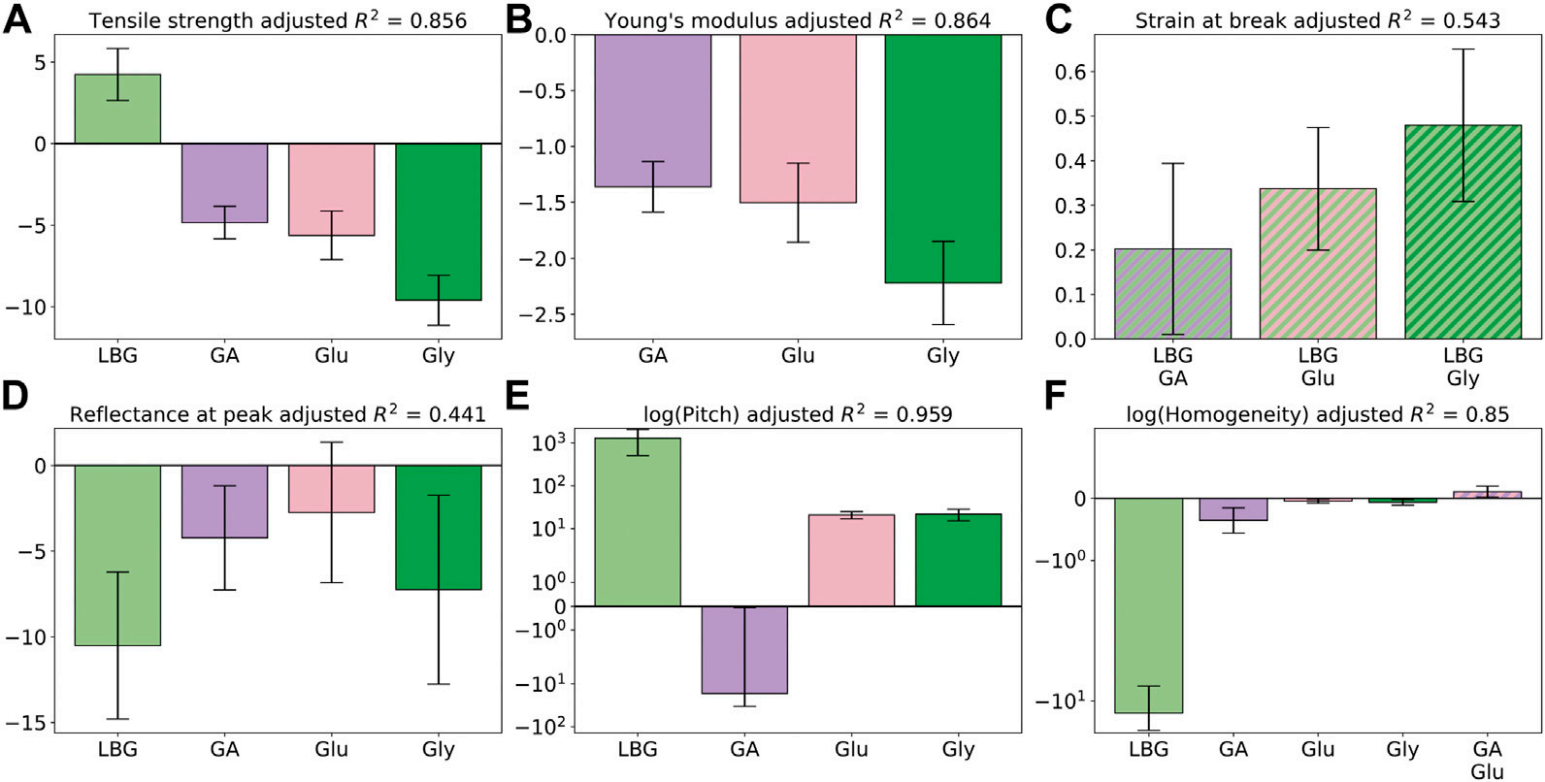
Bar plots of the multiple linear regression coefficients with standard errors calculated at 95%. Regression coefficients from tensile properties using data from 44 films: tensile strength index **(A)**, Young’s modulus **(B)**, and strain at break **(C)**. Regression coefficients from optical properties: reflectance at 550 nm using data from 56 films **(D)**, chiral nematic pitch using data from 13 films **(E)**, and chiral nematic homogeneity from 13 films **(F)**. Note the logarithmic scales in **(E)** and **(F)**, and that non-linear interactions between the indicated additives are shown by stripes.

Supplementary Equations S1–S6 give the equations resulting from the multiple linear regressions used to predict quantitative properties based on additive concentrations, with Figure 5 showing the values of the regression coefficients for tensile and optical parameters. Based on the adjusted $R^{2}$ values (shown in Figure 5), it is evident that some properties were better modelled than others. The properties with low adjusted $R^{2}$ values are the optical results, reflectance and total transmittance, and the strain at break. The low adjusted $R^{2}$ for the strain at break regression is attributed to the large variability in the breaking point of these films (see representative stress-strain curves shown Figure 3). The high standard deviation may be related to the overall brittleness of the films, which increases the likelihood of small cracks or fractures introduced in the die-cutting process. Conversely, the low adjusted $R^{2}$ values for the optical regressions are related to the very similar optical response of most films, except for a few outliers that presented distinct optics, such as chiral nematic reflection. The regressions related to chiral nematicity only considered a fraction of the films produced in this work (13 out of 81 were chiral nematic). Specifically, only one LBG-containing film was included in the analysis, and this film did not contain any other additives, leading to LBG coefficients in the homogeneity and pitch regressions determined from a single fit and severely limiting the strength of any conclusions drawn from these regressions.

Apart from these properties, the adjusted $R^{2}$ values indicate good model fits with high predictability. All mechanical strength properties are reduced by the addition of glucuronic acid, glucose, and glycerol, results that correspond well to the literature (He et al., 2018), whereas LBG improved the tensile strength index but had no effect on the Young’s modulus. Finally, all additives had a negative effect on the homogeneity of the chiral nematic structure, with the effects of glucose and glycerol similar in magnitude, glucuronic acid about an order of magnitude larger, and LBG about another order of magnitude larger (see comment above related to chiral nematic regressions). To better predict additive/additive combination effects on chiral nematic structure, more chiral nematic films need to be produced and fed into the model.

Finally, most interaction coefficients in the regressions with high adjusted $R^{2}$ values were not significantly separated from zero, indicating that the effect of any combination of these additives can be approximated by the sum of their parts. This may be important for the purpose of future testing as it implies that these additives can be screened using smaller experimental designs optimized for linear effects.

### 3.4 Qualitative models

As discussed above, POM images were used to interpret film order, along a hypothesized continuum from a twisted chiral nematic structure (*CN*) to an untwisted planar structure (*UTP*), with an intermediate small domain (*SD*) state in between. The defined order categories were then modelled using a logistic regression and a random forest based on the concentration of additives in the films.

**FIGURE 6:**
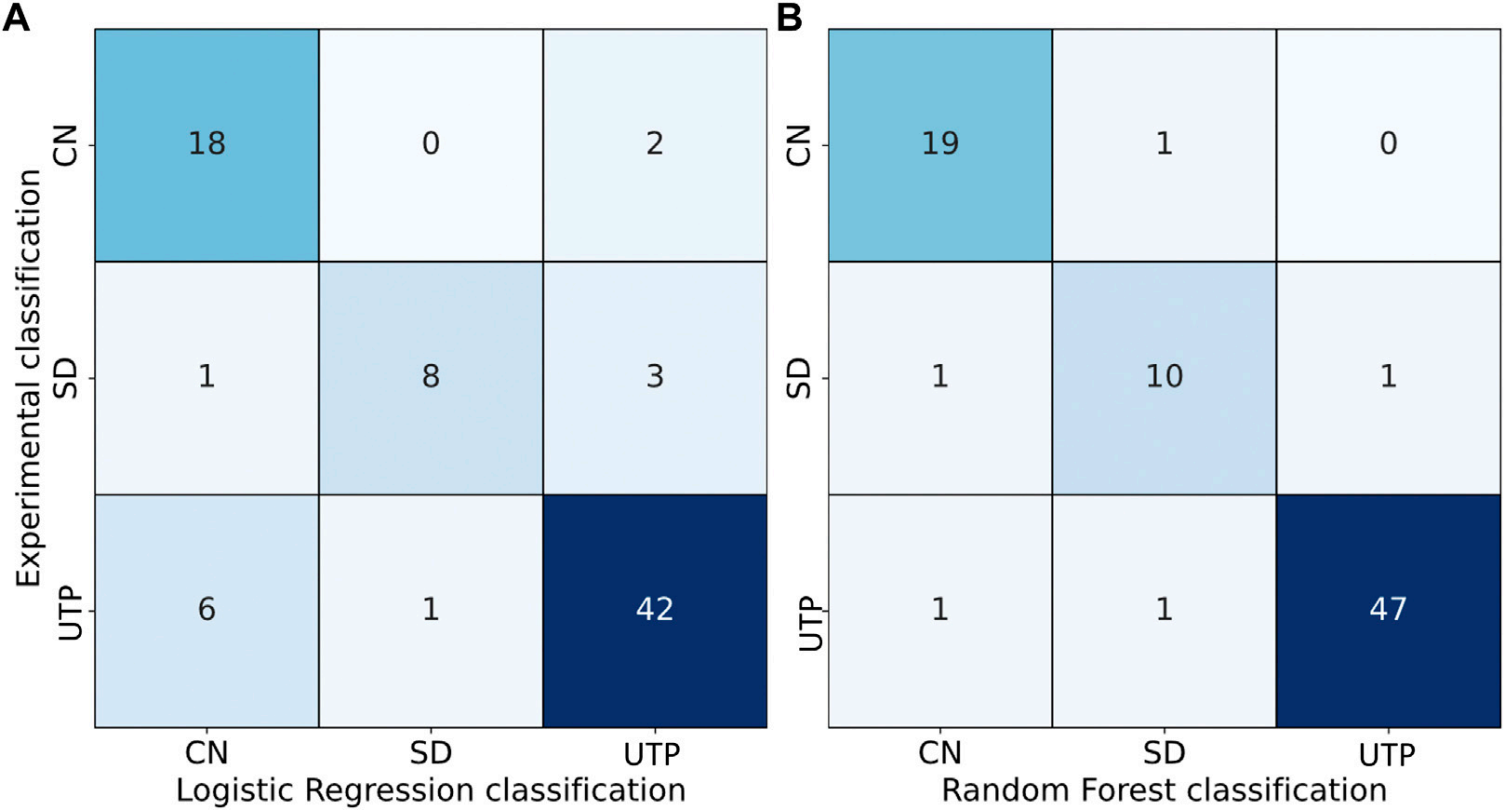
Confusion matrices showing POM-based film categories on the *y*-axis with logistic regression **(A)** and random forest predictions **(B)** on the *x*-axis. The correctly predicted samples lie along the diagonal, whereas off-diagonal entries are misclassifications.

The POM categories were modelled by a logistic regression (smaller model) and a random forest classifier (larger model). The two different models were used to test whether a larger model could capture phenomena neglected by the smaller model. Both models were constructed from the additive concentrations and the interaction terms of 81 films. These models were then validated using a 5-fold cross validation and the resulting confusion matrices are presented in Figure 6 and the POM categorization is presented in Supplementary Tables S1–S5.

Of the 10 films with a LBG content in the range of 1–2 wt%, five were misclassified as *CN* instead of *UTP* by the logistic regression but not by the random forest. The reason is clarified upon closer inspection of the film compositions and their class designations, as films within this concentration range shift between *CN* and *UTP* with very small changes in additive concentration. This may signal a transition point where LBG adsorption begins to interfere with the chiral interactions between CNCs. The random forest classifier seems to perform better in this transition regime, suggesting that the underlying phenomenon is more complex than can be captured with a simple logistic regression. Generally, the random forest performed slightly better in predicting all classifications, but both models are reasonably predictive, considering the potential error sources that can occur along the film making process.

**FIGURE 7:**
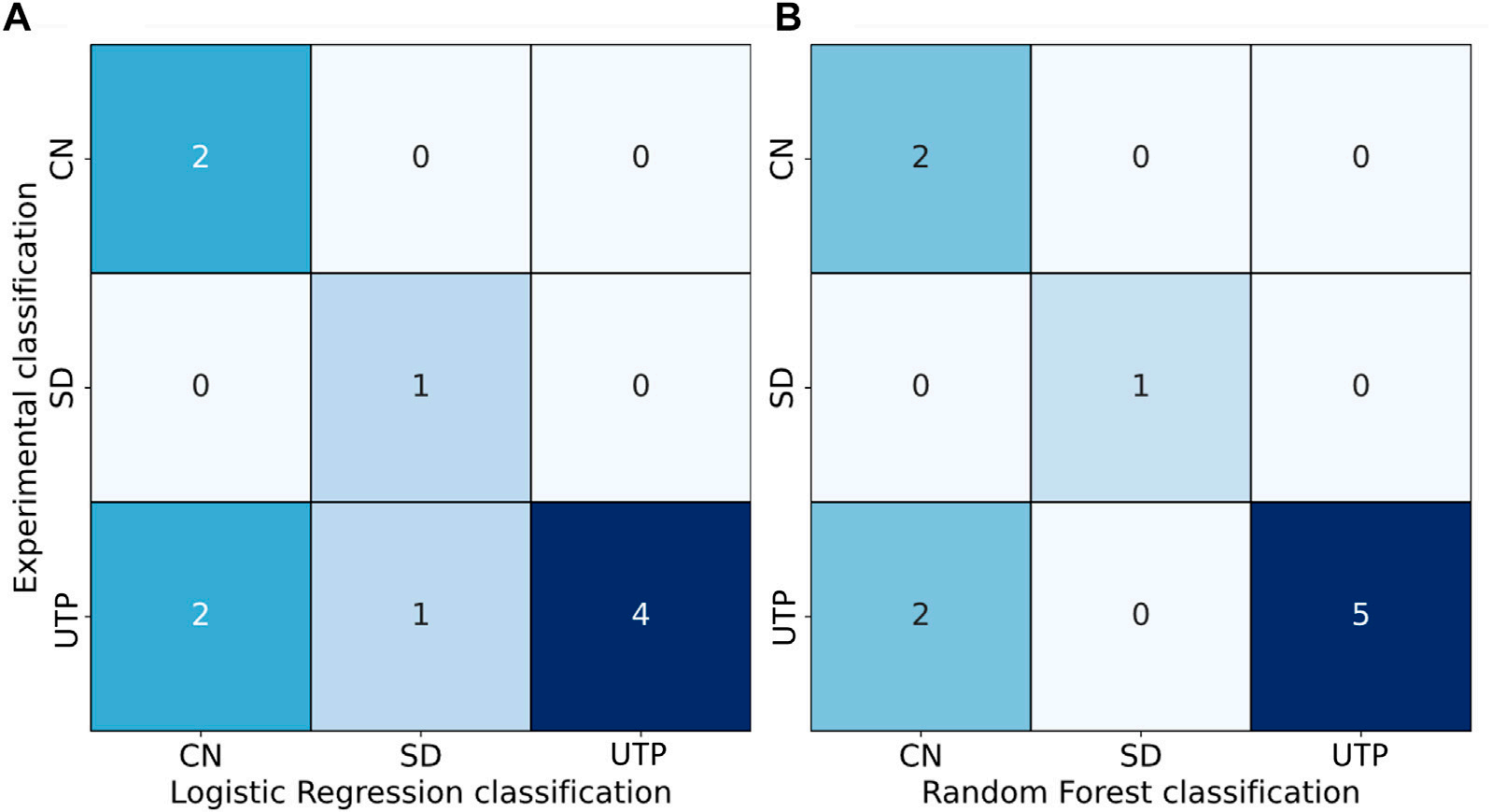
Confusion matrices showing POM-based film categories on the *y*-axis and logistic regression **(A)** and random forest predictions **(B)** from ten test films not seen by the model on the *x*-axis. The correctly predicted samples lie along the diagonal, whereas off-diagonal entries are misclassifications.

To test the regression models, new films (Supplementary Table S6) were prepared that were not used in the training of the models. The resulting confusion matrices are presented in Figure 7.

These matrices are like the confusion matrices presented in Figure 6, also with some wrongly predicted *UTP* films in the logistic regression, indicating that the issue of the transition region persists even when all films are used to define the model. Furthermore, the similarity indicates that the test samples reasonably represent the training samples and support the conclusions of the 5-fold cross validation.

**FIGURE 8:**
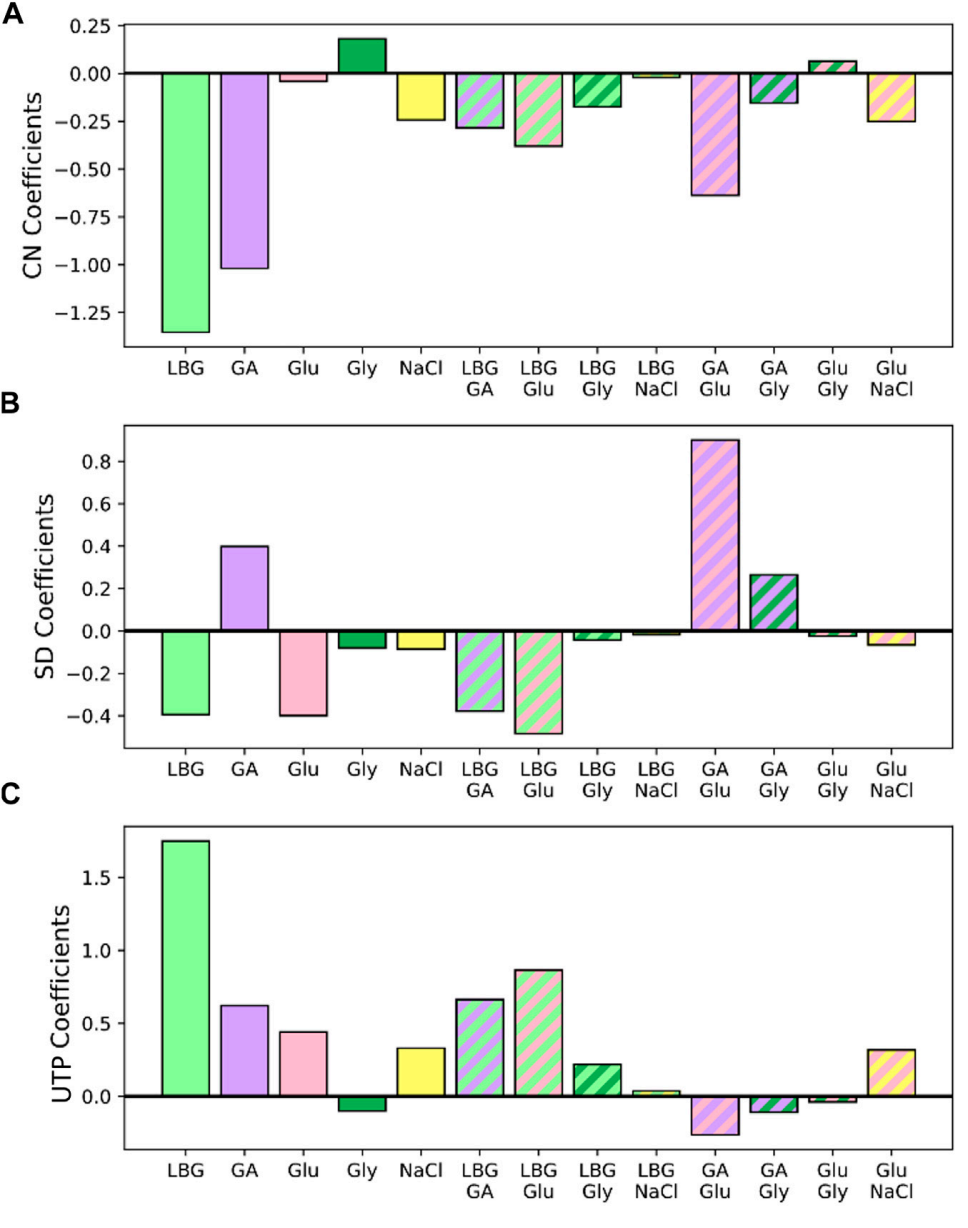
Coefficients of the logistic regressions that predict whether a film is chiral nematic (CN) **(A)**, has small domain (SD) organization **(B)**, or is untwisted planar (UTP) **(C)**.

Next, by analyzing the coefficients of the logistic regressions, correlations between additives and the different POM categories were identified. The resulting coefficients of this model are shown in Figure 8.

The *CN* coefficients for both LBG and glucuronic acid are large and negative, and all interaction coefficients that contain either of these two additives are negative (Figure 8A). This can be interpreted as LBG and glucuronic acid interfering with the formation of a chiral nematic structure. The *CN* coefficients for glucose, glycerol, and their interactions, are all small, indicating that these additives/additive combinations do not significantly interfere with chiral nematic phase formation, consistent with the results of others (Mu and Gray, 2014; Xu et al., 2018), but also that the addition of glucose and/or glycerol does not induce chiral nematic organization in films that would otherwise not organize in this way.

Regarding *SD* (Figure 8B), positive coefficients are obtained for glucuronic acid and its interactions with glucose and glycerol. Since the glucose and glycerol coefficients are negative, the effects of glucuronic acid are interpreted as dominant. This is very unlike the behavior of organic acids of similar size used in another work, where even films with a high concentration retained their chiral nematic organization (Li et al., 2021). The reason for this discrepancy could be that the CNCs used by Li et al. (2021). were in acidic form, resulting in a higher association of protons to the organic acids as the suspension is concentrated. LBG and all its interactions are negative, indicating that only very low LBG quantities can be tolerated to achieve a film with small domain microstructural organization.

Finally, for the *UTP* classification, all additives except glycerol and glucose/glucuronic acid interactions have a positive coefficient, but LBG has the strongest effect by far, which agrees with LBG adsorption interfering with chiral interactions and hastening gelation. For the remaining coefficients, all additives except glycerol seem to prevent chiral nematic assembly at sufficiently high concentrations. Thus, within the tested concentration range (up to 20 wt% glycerol), chiral nematic organization is not prevented by glycerol, which is consistent with the literature on CNC-glycerol films (Xu et al., 2018).

That all interactions with LBG prevent chiral nematic organization (films are predominately classified as *UTP*) and that all interactions with glucuronic acid (except in the presence of LBG) lead to the small domain organization points to a hierarchy of additive effects. Specifically, the effects of LBG dominate over the effects of the other three additives, and the effects of glucuronic acid dominate over the effects of glucose and glycerol. This relates well to the general understanding that it is the CNCs, and not the additives, that drive the ordering in the films, so additives that directly influence CNC shape and colloidal properties are expected to dominate.

## 4 Discussion

A DoE approach was used to study the influence of LBG, glucuronic acid, glucose, and glycerol as biobased additives on the properties of CNC films. In total, 81 CNC films were cast at different levels and combinations of additives, to cover a large additive space (see Supplementary Tables S1–S7 for film compositions) and eventually enable composition-structure-property optimizations.

Glucose, glycerol, and LBG have been included in CNC suspensions and films previously, but to our knowledge not in mixed compositions, whereas we have found no reports using glucuronic acid. Glucose has been reported to redshift the chiral nematic pitch (Mu and Gray, 2014), glycerol to redshift and plasticize (Xu et al., 2018), and LBG to adsorb to CNC surfaces and inducing gelation (Hu et al., 2014). Specifically, Mu and Gray observed a redshift with glucose contents up to 48 wt%, which was interpreted as glucose hastening the onset of gelation due to an increase in viscosity owing to a decrease in water activity (Mu and Gray, 2014). Xu et al., (2018) observed redshifts to wavelengths up to 834 nm and a significant plasticizing effect with glycerol contents of 10–40 wt%, attributing the result to glycerol acting as a spacer. Although not directly addressed in the literature, we hypothesize that LBG adsorption may screen the chiral interactions between twisted CNC, whereas glucuronic acid is hypothesized to influence CNC self-assembly in a concentration-dependent manner, like other organic acids (Li et al., 2021) or blue dextran (Beck-Candanedo et al., 2006).

In general, according to the models used in this work, glucuronic acid, glucose, and glycerol were found to behave similarly in terms of mechanical properties, lowering the Young’s modulus and increasing the tensile strength index. Conversely, the Young’s modulus was unaffected by LBG. None of the additives were found to significantly influence transparency, haze, or reflectance, with the exception of films which had chiral nematic reflections. Considering the chiral nematic films, glucose and glycerol additions redshifted the pitch. Glucuronic acid did not significantly shift the pitch but interfered with chiral nematic organization at concentrations above 3 wt%. LBG strongly redshifted the pitch at very low concentrations and prevented chiral nematic assembly already at 0.1 wt%. These observations are consistent with the above paragraph except for glucuronic acid whose influence was different from what has been reported with other organic acids. Most additives/additive combinations increased the width of the chiral nematic reflectance and/or decreased reflectance intensity.

The broad screening of additive concentrations performed in this study did not optimize for anything specific, but recorded many different results. This gives indications of certain regions in the concentration space where one could optimize for specific film properties, rather than examples of films fully optimized for certain outcomes. Since correlations between additives and their interactions, and certain film properties were found, these models can be used to predict concentration regions within which an optimization design could be used to create films tailored for certain applications.

Examples of these applications include packaging, where strength, flexibility, barrier properties, and optical properties are important (Wang et al., 2020). From our data, a tensile strength index of 49.7 Nm/g, a strain at break of 3.7%, and a transparency of 87% at 550 nm can be achieved with a combination of 20 wt% LBG and 10 wt% glycerol (N14), although further studies are needed to assess the barrier properties of these films to determine their suitability for packaging. Another potential application is as biobased solar cell substrates, where optical and mechanical performance is important (Zhou et al., 2013). From our data, 10 wt% LBG and 20 wt% glucose (N10) produces transparent films (88% at 550 nm) with a strain at break of 6.7%, compared to that of pure CNC films (N01, N02), which have a transparency of 88% and a strain a at break of 0.57%. Another possible application is in IR reflection for smart windows or solar cells, where a tunable, uniform, chiral nematic structure can be used to reflect heat (Khandelwal et al., 2014). By analyzing the linear regressions for homogeneity and pitch, we can determine conditions that redshift the pitch with minimal reductions in homogeneity, for instance using either glucose or glycerol. Among the films studied in this work, the best choice for this application is the film containing 20 wt% glycerol (N27), which had a pitch of 820 nm and a homogeneity of 50% that of the pure CNC film, which had a pitch at 600 nm.

Going forward, by combining the types of optimizations based on the quantitative and qualitative models demonstrated in this work, a concentration space that results exclusively in chiral nematic films can be defined. According to our data, this space is larger for glycerol compared to glucose, followed by glucuronic acid, and LBG with the smallest space, again reflective of a hierarchy in additive effects. As many of the additives explored in this work were found to decrease the homogeneity of the chiral nematic structure, a similar approach could be used to determine the redshift/homogeneity trade-off in other additives. This type of endeavor could initially be streamlined by not taking additive interactions into account, as we have found little evidence that additive interactions play an important role, at least for the current additive set. An example of an additive that would be interesting to investigate for these kinds of applications is dextran, which according to Adstedt et al. (2020) only increases the peak width slightly despite redshifts at all loadings tested (5%–38%). Finally, similar optimizations could be done for blueshifts, toughness, $\varepsilon_{b}$ , and any other parameter, provided a reliable quantification approach is at hand.

## 5 Conclusion

In this work, we aimed to understand whether data-based modelling tools could be used to predict mechanical and optical film outcomes in CNC films containing multiple additives. Generally, the modelling tools applied in this study provided reasonable predictive power and could be used to shed light on the dominant interactions at play in complex compositions. For parameters that produced regression coefficients from multiple linear regressions with high adjusted *R* ^2^ values, interactions between additives were limited, indicating the validity of the model choice. Logistic regressions showed promise in the prediction of the state of assembly in CNC films based on additive content, which may prove useful for tailoring film compositions for specific optical outcomes. Finally, the approach suggested in this work provides a framework for modeling CNC films which can be used to identify application-specific film compositions to accelerate real world usage of these films.

## Data Availability

The original contributions presented in the study are included in the article/Supplementary Material, further inquiries can be directed to the corresponding author.
