# Supplementary material for: Design of experiments to investigate multi-additive cellulose nanocrystal films
Source: Front Mol Biosci. 2022 Nov 4;9:988600. doi: 10.3389/fmolb.2022.988600 (PMC9673984; doi:10.3389/fmolb.2022.988600)
Supplement: Supplementary file 1 [file Table1.DOCX]

Supplementary Information

**Table S1.** First experimental design to screen additives effects.

| **Exp Name** | **LBG** | **GlucAc** | **Glucose** | **Glycerol** | **CNC** | **Category** |
| --- | --- | --- | --- | --- | --- | --- |
| N01 | 0 | 0 | 0 | 0 | 1 | CN |
| N02 | 0 | 0 | 0 | 0 | 1 | CN |
| N03 | 0.2 | 0 | 0 | 0 | 0.8 | UTP |
| N04 | 0 | 0.2 | 0 | 0 | 0.8 | SD |
| N05 | 0 | 0 | 0.2 | 0 | 0.8 | CN |
| N06 | 0.18 | 0.09 | 0 | 0 | 0.72 | UTP |
| N08 | 0.09 | 0.18 | 0 | 0 | 0.73 | UTP |
| N09 | 0.19 | 0 | 0.11 | 0 | 0.70 | UTP |
| N10 | 0.09 | 0 | 0.19 | 0 | 0.71 | UTP |
| N11 | 0 | 0.2 | 0.1 | 0 | 0.7 | SD |
| N12 | 0 | 0.2 | 0.1 | 0 | 0.7 | SD |
| N13 | 0.10 | 0 | 0 | 0.18 | 0.72 | UTP |
| N14 | 0.19 | 0 | 0 | 0.10 | 0.72 | UTP |
| N16 | 0 | 0.19 | 0 | 0.10 | 0.71 | SD |
| N18 | 0 | 0.10 | 0 | 0.21 | 0.69 | SD |
| N21 | 0 | 0 | 0 | 0.14 | 0.86 | CN |
| N22 | 0.16 | 0 | 0.13 | 0 | 0.70 | UTP |
| N24 | 0 | 0 | 0.13 | 0 | 0.87 | CN |
| N25 | 0 | 0.13 | 0 | 0 | 0.87 | SD |
| N26 | 0.13 | 0 | 0 | 0 | 0.87 | UTP |
| N27 | 0 | 0 | 0 | 0.20 | 0.80 | CN |
| N28 | 0 | 0 | 0.10 | 0.19 | 0.71 | CN |
| N31 | 0 | 0.1 | 0.2 | 0 | 0.7 | SD |
| N32 | 0 | 0.1 | 0.2 | 0 | 0.7 | SD |
| N36 | 0.07 | 0.07 | 0.07 | 0.07 | 0.70 | UTP |
| N37 | 0.06 | 0.06 | 0.06 | 0.07 | 0.74 | UTP |
| N38 | 0.06 | 0.07 | 0.07 | 0.07 | 0.74 | UTP |
| N39 | 0.07 | 0.07 | 0.07 | 0.07 | 0.73 | UTP |

**Table S2.** Second design to test the limits of effects from glucuronic acid and locust bean gum.

| **Exp Name** | **LBG** | **GlucAc** | **Glucose** | **Glycerol** | **CNC** | | **Category** |
| --- | --- | --- | --- | --- | --- | --- | --- |
| N50 | 0 | 0 | 0 | 0 | | 1 | CN |
| N51 | 0 | 0.41 | 0 | 0 | | 0.59 | UTP |
| N52 | 0 | 0.25 | 0 | 0 | | 0.75 | UTP |
| N53 | 0 | 0 | 0 | 0 | | 1 | CN |
| N54 | 0 | 0.31 | 0 | 0 | | 0.69 | UTP |
| N55 | 0 | 0.35 | 0 | 0 | | 0.65 | UTP |
| N56 | 0.03 | 0 | 0 | 0 | | 0.97 | UTP |
| N57 | 0.02 | 0 | 0 | 0 | | 0.98 | UTP |
| N58 | 0.01 | 0 | 0 | 0 | | 0.99 | SD |
| N59 | 0.001 | 0 | 0 | 0 | | 0.999 | CN |
| N60 | 0.03 | 0.10 | 0 | 0 | | 0.87 | UTP |
| N61 | 0.02 | 0.10 | 0 | 0 | | 0.89 | UTP |
| N62 | 0.010 | 0.10 | 0 | 0 | | 0.89 | UTP |
| N63 | 0.001 | 0.1 | 0 | 0 | | 0.90 | SD |
| N64 | 0 | 0.03 | 0 | 0 | | 0.97 | CN |
| N65 | 0 | 0.02 | 0 | 0 | | 0.98 | CN |
| N66 | 0 | 0.01 | 0 | 0 | | 0.99 | CN |
| N67 | 0 | 0.001 | 0 | 0 | | 0.999 | CN |

**Table S3.** Third design to test model existing at the time and to test the limits of LBG.

| **Exp Name** | **LBG** | **GlucAc** | **Glucose** | **Glycerol** | **CNC** | **Category** |
| --- | --- | --- | --- | --- | --- | --- |
| N69 | 0.02 | 0.02 | 0 | 0 | 0.96 | UTP |
| N70 | 0.09 | 0.11 | 0.12 | 0 | 0.68 | UTP |
| N71 | 0.05 | 0 | 0 | 0.16 | 0.79 | UTP |
| N72 | 0 | 0.05 | 0.05 | 0 | 0.90 | CN |
| N73 | 0.1 | 0.03 | 0 | 0.11 | 0.74 | UTP |
| N75 | 0.00675 | 0 | 0 | 0 | 0.99325 | CN |
| N76 | 0.009 | 0 | 0 | 0 | 0.991 | CN |
| N77 | 0.01125 | 0 | 0 | 0 | 0.98875 | CN |
| N78 | 0.0135 | 0 | 0 | 0 | 0.9865 | CN |

**Table S4.** Fourth design to test the effects of NaCl.

| **Exp Name** | **LBG** | **GlucAc** | **Glucose** | **Glycerol** | **CNC** | **NaCl** | **Category** |
| --- | --- | --- | --- | --- | --- | --- | --- |
| N80 | 0.01 | 0.019 | 0.10 | 0 | 0.87 | 0 | UTP |
| N81 | 0 | 0.07 | 0.12 | 0 | 0.80 | 0 | SD |
| N82 | 0.02 | 0.04 | 0.17 | 0 | 0.78 | 0 | UTP |
| N83 | 0.008 | 0.08 | 0.19 | 0 | 0.72 | 0 | UTP |
| N84 | 0.008 | 0.04 | 0.21 | 0 | 0.74 | 0 | UTP |
| N85 | 0 | 0 | 0.19 | 0 | 0.77 | 0.04 | UTP |
| N86 | 0.05 | 0 | 0.21 | 0 | 0.74 | 0.01 | UTP |
| N87 | 0.15 | 0 | 0.24 | 0 | 0.60 | 0.009 | UTP |
| N88 | 0.0008 | 0 | 0.09 | 0 | 0.88 | 0.02 | UTP |
| N89 | 0 | 0 | 0.09 | 0 | 0.91 | 0.008 | UTP |
| N91 | 0 | 0 | 0.01 | 0 | 0.89 | 0.013 | UTP |
| N92 | 0 | 0 | 0.29 | 0 | 0.70 | 0.015 | UTP |

**Table S5.** Fifth design to test the similarities between the effects of NaCl and glucuronic acid.

| **Exp Name** | **LBG** | **GlucAc** | **Glucose** | **Glycerol** | **NaCl** | **CNC** | **Category** |
| --- | --- | --- | --- | --- | --- | --- | --- |
| N103 | 0 | 0.02 | 0 | 0 | 0 | 0.98 | CN |
| N104 | 0.07 | 0.04 | 0.1 | 0 | 0 | 0.79 | UTP |
| N105 | 0.002 | 0.17 | 0 | 0 | 0 | 0.83 | UTP |
| N602 | 0.03 | 0.1 | 0 | 0 | 0 | 0.87 | UTP |
| N672 | 0.02 | 0 | 0 | 0 | 0 | 0.98 | UTP |
| N832 | 0.008 | 0.08 | 0.2 | 0 | 0 | 0.72 | UTP |
| N802 | 0.01 | 0.02 | 0.1 | 0 | 0 | 0.87 | UTP |
| N812 | 0 | 0.08 | 0.12 | 0 | 0 | 0.8 | SD |
| N842 | 0.008 | 0.04 | 0.21 | 0 | 0 | 0.74 | UTP |
| N852 | 0 | 0 | 0.2 | 0 | 0.036 | 0.76 | UTP |
| N882 | 0.0007 | 0 | 0.1 | 0 | 0.023 | 0.88 | UTP |
| N892 | 0 | 0 | 0.03 | 0 | 0.006 | 0.96 | CN |
| N108 | 0.05 | 0 | 0.2 | 0 | 0.036 | 0.71 | UTP |
| N109 | 0.02 | 0 | 0 | 0 | 0.023 | 0.96 | UTP |

**Table S6.** Design to test the predictive power of the model.

| **Exp Name** | **LBG** | **GlucAc** | **Glucose** | **Glycerol** | **CNC** | **NaCl** |
| --- | --- | --- | --- | --- | --- | --- |
| T01 | 0 | 0 | 0.18 | 0.13 | 0.7 | 0 |
| T02 | 0.03 | 0 | 0 | 0.14 | 0.83 | 0 |
| T03 | 0 | 0.044 | 0.22 | 0.044 | 0.69 | 0 |
| T04 | 0 | 0 | 0.14 | 0 | 0.84 | 0.018 |
| T05 | 0 | 0 | 0.22 | 0 | 0.76 | 0.02 |
| T06 | 0.027 | 0 | 0 | 0 | 0.93 | 0.05 |
| T07 | 0.001 | 0 | 0 | 0.16 | 0.83 | 0.009 |
| T08 | 0 | 0 | 0 | 0.24 | 0.76 | 0 |
| T09 | 0.009 | 0.16 | 0.18 | 0 | 0.66 | 0 |
| T10 | 0 | 0 | 0 | 0 | 0.96 | 0.04 |

**Table S7.** Pre-study concentrations.

| **Exp Name** | **Additive** | **Additive conc. (%)** |
| --- | --- | --- |
| T29 | GK | 5 |
| T30 | GK | 20 |
| T31 | GK | 25 |
| T32 | GK | 30 |
| T33 | Guar Gum | 10 |
| T34 | CMC | 10 |
| T35 | LBG | 10 |
| T36 | Glucose | 10 |
| T37 | GK | 10 |
| T38 | PVA | 10 |
| T39 | Dextran | 10 |
| T40 | Pectin | 10 |
| T41 | N/A | 0 |
| T42 | Glucose | 5 |
| T43 | Glucose | 20 |
| T44 | Glucose | 25 |
| T45 | Glucose | 30 |
| T46 | GK | 5 |
| T47 | GK filtered | 10 |
| T49 | GK filtered | 30 |
| T50 | 10% Glu 5% gly | 15 |
| T51 | 10% Glu 1% gly | 11 |
| T52 | 10% Glu 10% gly | 20 |
| T53 | 10% GK 5%gly | 15 |
| T54 | 10% GK 1%gly | 11 |
| T55 | 10% GK 10%gly | 20 |

**Table S8.** Measured optical and mechanical properties of all samples. N/A indicates that the metric was not applicable for the given sample and N/M indicates a parameter that was not measured. T refers to transmittance and pitch refers to the maximum reflection wavelength.

| **Exp Name** | **Category** | **CN**  **(Y/N)** | **Young’s Modulus**  **(*MN/***$\boldsymbol{m}^{\boldsymbol{2}}$***)*** | **Strain At Break**  **(%)** | **Tensile Strength Index**  ***(Nm/g)*** | **T at 550 nm** | **Pitch**  **(nm)** |
| --- | --- | --- | --- | --- | --- | --- | --- |
| **N01** | CN | Y | 15.95 | 0.57 | 48.54 | 0.74 | 601 |
| **N02** | CN | Y | 16.23 | 0.53 | 56.24 | 0.70 | 586 |
| **N03** | UTP | N | 14.16 | 1.53 | 74.20 | 0.90 | N/A |
| **N04** | SD | N | 8.88 | 0.91 | 35.72 | 0.89 | N/A |
| **N05** | CN | Y | 8.91 | 1.22 | 29.78 | 0.88 | 787 |
| **N06** | UTP | N | 12.22 | 0.91 | 55.44 | 0.89 | N/A |
| **N08** | UTP | N | 10.31 | 2.21 | 48.75 | 0.88 | N/A |
| **N09** | UTP | N | 11.01 | 1.47 | 63.91 | 0.90 | N/A |
| **N10** | UTP | N | 5.78 | 6.75 | 28.79 | 0.90 | N/A |
| **N11** | SD | N | 9.01 | 1.56 | 37.39 | 0.89 | N/A |
| **N12** | SD | N | 6.55 | 1.24 | 25.17 | 0.89 | N/A |
| **N13** | UTP | N | 5.84 | 4.44 | 24.41 | 0.90 | N/A |
| **N14** | UTP | N | 9.89 | 3.65 | 49.72 | 0.89 | N/A |
| **N16** | SD | N | 4.88 | 1.29 | 15.36 | 0.86 | N/A |
| **N18** | SD | N | N/M | N/M | N/M | N/M | N/A |
| **N21** | CN | Y | 8.76 | 0.47 | 23.80 | 0.86 | 728 |
| **N22** | UTP | N | 8.49 | 2.46 | 45.91 | 0.87 | N/A |
| **N24** | CN | Y | 12.65 | 1.00 | 39.79 | 0.88 | 705 |
| **N25** | SD | N | 13.88 | 0.56 | 38.71 | 0.86 | N/A |
| **N26** | UTP | N | 13.28 | 1.57 | 72.57 | 0.90 | N/A |
| **N27** | CN | Y | 4.59 | 0.42 | 10.29 | 0.89 | 820 |
| **N28** | CN | Y | 4.09 | 2.39 | 9.64 | 0.69 | 917 |
| **N31** | SD | N | 5.99 | 1.77 | 25.47 | 0.88 | N/A |
| **N32** | SD | N | 5.93 | 1.26 | 20.70 | 0.90 | N/A |
| **N36** | UTP | N | 6.04 | 4.69 | 26.86 | 0.88 | N/A |
| **N37** | UTP | N | 6.95 | 3.04 | 28.12 | 0.90 | N/A |
| **N38** | UTP | N | 10.83 | 5.18 | 54.24 | 0.69 | N/A |
| **N39** | UTP | N | 6.99 | 3.59 | 29.36 | 0.89 | N/A |
| **N40** | UTP | N | 8.22 | 1.31 | 40.53 | 0.90 | N/A |
| **N41** | UTP | N | 8.38 | 1.31 | 41.03 | 0.90 | N/A |
| **N43** | UTP | N | 6.16 | 3.69 | 26.44 | 0.90 | N/A |
| **N44** | UTP | N | 7.74 | 5.37 | 36.85 | 0.87 | N/A |
| **N45** | UTP | N | 9.03 | 2.23 | 42.26 | 0.88 | N/A |
| **N46** | UTP | N | 7.26 | 3.73 | 34.21 | 0.86 | N/A |
| **N47** | UTP | N | 4.77 | 10.06 | 19.41 | 0.87 | N/A |
| **N48** | UTP | N | 3.38 | 7.60 | 15.06 | 0.87 | N/A |
| **N49** | UTP | N | 3.80 | 7.94 | 15.54 | 0.89 | N/A |
| **N51** | UTP | N | 3.45 | 2.43 | 12.47 | 0.91 | N/A |
| **N52** | UTP | N | 6.40 | 1.23 | 24.66 | 0.90 | N/A |
| **N54** | UTP | N | 4.45 | 2.05 | 18.81 | 0.90 | N/A |
| **N55** | UTP | N | 4.10 | 1.72 | 16.26 | 0.90 | N/A |
| **N56** | UTP | N | 13.56 | 1.14 | 62.49 | 0.90 | N/A |
| **N57** | UTP | N | 14.29 | 1.03 | 58.88 | 0.90 | N/A |
| **N58** | SD | N | 13.09 | 0.77 | 56.44 | 0.87 | N/A |
| **N59** | CN | Y | 15.55 | 0.55 | 46.26 | 0.82 | 658 |
| **N60** | UTP | N | 10.59 | 1.31 | 51.66 | 0.90 | N/A |
| **N61** | UTP | N | 10.69 | 1.33 | 54.12 | 0.90 | N/A |
| **N62** | UTP | N | 10.25 | 0.95 | 48.11 | 0.90 | N/A |
| **N63** | SD | N | 14.65 | 0.61 | 39.49 | 0.86 | N/A |
| **N64** | CN | Y | 14.09 | 0.71 | 55.08 | 0.82 | 554 |
| **N65** | CN | Y | 12.70 | 0.46 | 42.04 | 0.73 | 564 |
| **N66** | CN | Y | 16.70 | 0.50 | 42.31 | 0.70 | 565 |
| **N67** | CN | Y | 14.15 | 0.73 | 60.67 | 0.76 | 609 |
| **N69** | UTP | N | N/M | N/M | N/M | N/M | N/A |
| **N70** | UTP | N | N/M | N/M | N/M | N/M | N/A |
| **N71** | UTP | N | N/M | N/M | N/M | N/M | N/A |
| **N72** | CN | Y | N/M | 0.96 | 47.71 | 0.77 | 612 |
| **N73** | UTP | N | N/M | N/M | N/M | N/M | N/A |
| **N75** | CN | Y | N/M | N/M | N/M | N/M | N/A |
| **N76** | CN | Y | N/M | N/M | N/M | N/M | N/A |
| **N77** | CN | Y | N/M | N/M | N/M | N/M | N/A |
| **N78** | CN | Y | N/M | N/M | N/M | N/M | N/A |
| **N80** | UTP | N | N/M | N/M | N/M | N/M | N/A |
| **N81** | SD | N | N/M | N/M | N/M | N/M | N/A |
| **N82** | UTP | N | N/M | N/M | N/M | N/M | N/A |
| **N83** | UTP | N | N/M | N/M | N/M | N/M | N/A |
| **N84** | UTP | N | N/M | N/M | N/M | N/M | N/A |
| **N85** | UTP | N | N/M | N/M | N/M | N/M | N/A |
| **N86** | UTP | N | N/M | N/M | N/M | N/M | N/A |
| **N87** | UTP | N | N/M | N/M | N/M | N/M | N/A |
| **N88** | UTP | N | N/M | N/M | N/M | N/M | N/A |
| **N89** | UTP | N | N/M | N/M | N/M | 0.87 | N/A |
| **N91** | UTP | N | N/M | N/M | N/M | N/M | N/A |
| **N92** | UTP | N | N/M | N/M | N/M | N/M | N/A |
| **N93** | SD | N | N/M | N/M | N/M | N/M | N/A |
| **N97** | UTP | N | N/M | N/M | N/M | N/M | N/A |
| **N98** | UTP | N | N/M | N/M | N/M | N/M | N/A |
| **N100** | UTP | N | N/M | N/M | N/M | 0.85 | N/A |
| **N101** | UTP | N | N/M | N/M | N/M | 0.47 | N/A |
| **N103** | CN | Y | N/M | N/M | N/M | 0.78 | 631 |
| **N104** | UTP | N | N/M | N/M | N/M | N/M | N/A |
| **N105** | UTP | N | N/M | N/M | N/M | N/M | N/A |
| **N602** | UTP | N | N/M | N/M | N/M | N/M | N/A |
| **N672** | UTP | N | N/M | N/M | N/M | N/M | N/A |
| **N832** | UTP | N | N/M | N/M | N/M | N/M | N/A |
| **N802** | UTP | N | N/M | N/M | N/M | N/M | N/A |
| **N812** | SD | N | N/M | N/M | N/M | N/M | N/A |
| **N842** | UTP | N | N/M | N/M | N/M | N/M | N/A |
| **N852** | UTP | N | N/M | N/M | N/M | N/M | N/A |
| **N882** | UTP | N | N/M | N/M | N/M | N/M | N/A |
| **N892** | CN | Y | N/M | N/M | N/M | N/M | N/M |
| **N108** | UTP | N | N/M | N/M | N/M | N/M | N/A |
| **N109** | UTP | N | N/M | N/M | N/M | N/M | N/A |
| **N110** | UTP | N | N/M | N/M | N/M | N/M | N/A |
| **T36** | CN | Y | 14.51 | 0.89 | 43.10 | 0.85 | 727 |
| **T37** | CN | Y | 15.92 | 0.66 | 44.13 | 0.67 | 464 |
| **T38** | CN | Y | 19.00 | 0.53 | 45.92 | 0.71 | 611 |
| **T39** | CN | Y | 16.47 | 0.19 | 20.01 | 0.84 | 718 |
| **T41** | CN | Y | 20.18 | 0.60 | 54.57 | 0.77 | 647 |
| **T46** | CN | Y | 13.33 | 0.69 | 63.82 | 0.72 | 496 |
| **T47** | CN | Y | 14.87 | 0.77 | 62.24 | 0.77 | 470 |
| **T48** | N/M | N | N/M | N/M | N/M | 0.82 | N/A |
| **T49** | N/M | N | 12.22 | 1.08 | 61.15 | 0.87 | N/A |
| **T51** | CN | Y | 12.28 | 1.03 | 53.04 | 0.84 | 701 |
| **T53** | CN | Y | 14.04 | 1.03 | 65.65 | 0.73 | 458 |
| **T54** | CN | Y | 13.53 | 0.61 | 49.61 | 0.75 | 434 |
| **T55** | CN | Y | 14.55 | 0.73 | 58.73 | 0.79 | 420 |

**Table S9.** Average measured thicknesses, based on 5 measurements at different places on the films.

| **Exp Name** | **Thickness (µm)** |
| --- | --- |
| **N01** | 19.6 |
| **N02** | 23 |
| **N03** | 17.8 |
| **N04** | 18.4 |
| **N05** | 21.6 |
| **N06** | 23 |
| **N08** | 20.6 |
| **N09** | 22.4 |
| **N10** | 28.4 |
| **N11** | 20.2 |
| **N12** | 21.6 |
| **N13** | 23.8 |
| **N14** | 25 |
| **N16** | 19.6 |
| **N18** | 18.25 |
| **N21** | 21.8 |
| **N22** | 27 |
| **N24** | 22 |
| **N25** | 18.6 |
| **N26** | 21.6 |
| **N27** | 21.25 |
| **N28** | 25 |
| **N31** | 22.8 |
| **N32** | 19.5 |
| **N36** | 24.2 |
| **N37** | 20 |
| **N38** | 19.2 |
| **N39** | 18.4 |
| **N40** | 25.6 |
| **N41** | 26 |
| **N42** | N/M |
| **N43** | 27.4 |
| **N44** | 30 |
| **N45** | 26 |
| **N46** | 30 |
| **N47** | 33.6 |
| **N48** | 38.4 |
| **N49** | 33.6 |
| **N51** | 18.8 |
| **N52** | 18.8 |
| **N54** | 21.5 |
| **N55** | 18.8 |
| **N56** | 17.8 |
| **N57** | 17.8 |
| **N58** | 23 |
| **N59** | 19.8 |
| **N60** | 19 |
| **N61** | 20.8 |
| **N62** | 22.4 |
| **N63** | 18.6 |
| **N64** | 22.7 |
| **N65** | 25.3 |
| **N66** | 20.2 |
| **N67** | 23.7 |
| **N68** | 22.2 |
| **T36** | 34.4 |
| **T37** | 31.6 |
| **T38** | 32.1 |
| **T39** | 39.3 |
| **T41** | 33.9 |
| **T46** | 43.1 |
| **T47** | 35.3 |
| **T49** | 41.1 |
| **T51** | 40.3 |
| **T53** | 31.6 |
| **T54** | 31.7 |
| **T55** | 31.6 |

**Equations S1-6**. The fitted multiple linear regressions, in order from 1-6, tensile strength index (1), Young’s modulus (2), strain at break (3), reflectance (4), pitch (5) and homogeneity (6).

$$\begin{aligned} \sigma= 54.3+4.23c_{LBG}-4.83c_{GA}-5.62c_{Glu}-9.6c_{Gly} \#1 \end{aligned}$$

$$\begin{aligned} E=14.59-1.36c_{GA}-1.5c_{Glu}-2.21c_{Gly} \#2 \end{aligned}$$

$$\begin{aligned} \varepsilon_{b}= 1.18+{0.2c}_{LBG}c_{GA}-0.3c_{LBG}c_{Glu}-0.48c_{LBG}c_{Gly} \#3 \end{aligned}$$

$$\begin{aligned} R=11.84-11.63c_{LBG}-4.36c_{GA}-3.02c_{Glu}-7.3c_{Gly}\#4 \end{aligned}$$

$$\begin{aligned} pitch= 588+1293c_{LBG}-16.7c_{GA}-20.8c_{Glu}-21.8c_{Gly}\#5 \end{aligned}$$

$$\begin{aligned} H= 0.92-13c_{LBG}-0.37c_{GA}-0.036c_{Glu}-0.05c_{Gly}+0.11c_{GA}c_{Glu}\#6 \end{aligned}$$
